# Understanding the experiences and needs of individuals with Spinal Muscular Atrophy and their parents: a qualitative study

**DOI:** 10.1186/s12883-015-0473-3

**Published:** 2015-10-24

**Authors:** Ying Qian, Sarah McGraw, Jeff Henne, Jill Jarecki, Kenneth Hobby, Wei-Shi Yeh

**Affiliations:** SMA Foundation, 888 7th Ave #400, New York, NY 10106 USA; The Henne Group, 116 New Montgomery Street, Suite 812, San Francisco, California 94105 USA; Cure SMA, 925 Busse Rd, Elk Grove Village, IL 60007 USA; Biogen, 225 Binney Street, Cambridge, MA 02142 USA

**Keywords:** Spinal Muscular Atrophy (SMA), Patient, Parent, Diagnosis, Screening, Life impact, Qualitative

## Abstract

**Background:**

The clinical features of SMA, which range along a spectrum of severity, are relatively well described. In contrast, the literature on how individuals with SMA and their families experience this condition is limited. To address this gap, we undertook a qualitative study with individuals affected by SMA Types I, II and III, parents of those affected, and clinicians.

**Methods:**

We completed 16 focus group sessions and 37 interviews in the US with 96 participants including: 21 with individuals with SMA; 64 parents of individuals affected by SMA; and 11 clinicians who specialize in the care of SMA patients.

**Results:**

*The Diagnostic Journey*: Families reported substantial diagnostic delays owing to: 1) lack of awareness and knowledge about SMA; 2) the difficulty of distinguishing normal from abnormal development; and 3) the challenge of differential diagnosis. Lack of sensitivity in how clinicians communicated this potentially devastating diagnosis compounded parents’ negative impressions.

*Newborn Screening*: Parents generally held positive views about adding SMA to newborn screening panels. For example, it would: 1) enable earlier access to care; 2) shorten the diagnostic journey; and 3) give families more time to prepare to care for a disabled child. Some noted negative outcomes such as prematurely affecting a parent’s relationship with a child before symptoms are evident.

*The Psychosocial Impact of Living with SMA*: Ten thematic areas characterized the impact: 1) confronting premature death; 2) making difficult treatment choices; 3) fearing the loss of functional ability; 4) coming to terms with lost expectations; 5) loss of sleep and stress; 6) stigma; 7) limitations on social activities; 8) independence; 9) uncertainty and helplessness; and 10) family finances.

**Conclusions:**

The results of this study suggest high levels of burden experienced by individuals with SMA and their families. The difficulties of living with SMA begin with the long and often arduous process of finding a diagnosis for their child. Newborn screening for SMA is seen as an important step toward shortening this journey. The psychosocial effects of coping with SMA are substantial and wide ranging both for the individual living with this condition and family members of affected individuals.

## Background

Spinal muscular atrophy (SMA) is an autosomal recessive disorder; in almost 95 % of all cases, there is a homozygous deletion or mutation of the Survival Motor Neuron 1 gene (SMN1) [1, 2]. SMA is characterized by degeneration of alpha motor neurons in the spinal cord, resulting in progressive muscular weakness and atrophy [3–5]. Affecting approximately one in 10,000 live births, SMA is a leading fatal autosomal recessive disorder in infancy [6–8].

SMA is classified into three main phenotypes, stratified by age of onset and severity of disease [5]. Some experts use an expanded classification system that includes a fourth phenotype to distinguish adult onset SMA [5, 9]. The most severe form (Type I) represents approximately 60 % of incident SMA cases and symptoms usually present within six months of birth [5, 9, 10]. Individuals with this form generally experience severe muscle weakness and atrophy, and some develop difficulty swallowing. They usually require ventilatory support and most die before the age of two [11–13]. SMA Type II accounts for approximately 30 % of SMA cases. These patients generally experience less severe proximal muscle weakness than observed in Type I SMA, and symptom onset is relatively delayed, usually evident by 18 months of age. Patients often can sit unaided, but never gain the ability to walk. Other common characteristics of SMA Type II include impaired swallowing, breathing, and development of scoliosis [5,10] Many survive into adulthood, but life expectancy is shortened compared to the general population, often because of respiratory failure resulting from lung disease, which increases in severity with age [11].

Approximately 10 % of SMA cases are classified as SMA Type III [10]. SMA symptoms tend to be milder compared with Types I and II, appearing anytime between 18 months of age and adulthood [5]. These patients have a normal lifespan [5]. Patients may initially walk or stand unaided; however, as the patient ages, proximal muscle strength becomes progressively weaker, and patients might need assistance to stand or walk. Accordingly, after age 2, patients begin to experience frequent falls and fatigue [5, 11, 14, 15]. Type IV, the mildest form, is rare. Individuals with this form do not exhibit muscle weakness until adulthood [16].

Evidence suggests that earlier initiation of supportive therapies can improve outcomes [2, 17, 18]. Despite the fact that a genetic test is available for newborn screening (NBS) [11], diagnostic testing currently is performed only when a clinician recognizes symptoms, often delaying diagnosis [2, 19]. Beyond a single study in Australia, which noted the negative impact of diagnostic delays [20], little is known about how families arrive at a diagnosis of SMA, how delays effect the disease trajectory, or a how a delay impacts a family’s ability to care for their child.

SMA can be a devastating disease, yet there is limited knowledge on how the disease affects the lives of individuals and families. Some studies have indicated that patients and families experience a lower quality of life and higher levels of stress [21–24]. However, much of this literature is based on studies conducted outside of the United States and does not offer an in-depth examination of perceptions about life with this disease and the associated burdens.

Here we report on one part of a larger study to understand the effect of SMA on the lives of individuals with this condition and their families and what would constitute meaningful improvements or decrements in function. In this report, we examine: 1) Factors that affected how families arrived at a diagnosis, as described by parents and clinicians; 2) Parents’ views on newborn screening; and 3) The impact of SMA on the lives of individuals with SMA and their parents.

## Methods

This study employed qualitative methods to elicit the views and experiences of individuals with SMA, parents, and clinicians who care for SMA patients in their own words. From June to October 2014, we completed 16 focus groups: 7 in connection with the Cure SMA national convention and 9 across five clinic locations (Denver, Boston, Palo Alto, Cincinnati or Chicago). We chose the sites because they were linked to Cure SMA chapters with active memberships with leading SMA clinicians. In addition, we conducted 37 semi-structured telephone interviews with individuals with SMA, parents or clinicians who could not attend the focus groups.

To recruit participants, we sent email invitations to 1,052 SMA families and clinicians identified through Cure SMA mailing lists and supplemental e-mail blasts to members and chapter leaders of SMA advocacy organizations. Individuals were eligible for the study if they: a) had SMA Type I, II, or III b) were a parent of a child with SMA Type I, II or III, aged one to 25 years alive within the past two years; or c) were a clinician involved in SMA clinical trials and caring for SMA patients at least 20 hours per week.

Study participants 12 years of age or older signed an informed consent document, and those 11 years of age or younger signed an assent document prior to the start of an interview or focus group. We obtained parental consent for individuals under age 18 years. Study participants did not receive honoraria or any form of compensation. The Ethical & Independent Review Services institutional review board (IRB) approved the study protocol (Reference #: 14051–01).

A trained interview/moderator conducted the focus groups (60–90 minutes long) and telephone interviews (30–60 minutes) using a semi-structured guide (See Appendix). Each session was audio-recorded and transcribed for analysis. The questions covered ten domains: 1) obtaining a diagnosis; 2) views on newborn screening; 3) the impact of SMA on the individual and the family; 4) treatments and avoiding hospitalization; 5) respiratory care; 6) declines in motor function over the preceding twelve months; 7) meaningful changes in motor function; and 8) expectations about improvements resulting from treatment. For the final two domains participants were asked to comment on items of the Expanded Hammersmith Functional Motor Scale (HFMSE) and any improvements in function that were not already captured by this scale [25–27]. For the purposes of this publication, we focus on domains one, two and three; findings related to the remaining areas will be described in a future publication.

Consistent with grounded theory [28, 29] two analysts independently read the transcripts, identified core concepts, and then jointly created a start-list of codes with definitions. One analyst then applied the codes to text segments within each transcript and both analysts reviewed the coding and revised the code list as necessary. Two additional coders independently coded 10 percent of the transcriptions and any discrepancies between the initial coding and the secondary coding were reconciled. Finally, we identified unifying themes that characterized participants’ experiences and views. We employed *Dedoose* [30], a qualitative analysis software package, to facilitate the coding and management of text segments. Saturation analysis showed that no new concepts emerged in the final 10 percent of the interview/focus group sessions suggesting that we fully covered the range of salient topics within each of the ten domains [31].

## Results

### Study sample

A total of 136 individuals responded to the recruitment emails and were screened for eligibility, 126 individuals were eligible, and 96 individuals participated in the study. Of the 96, 21 were individuals with SMA who were approximately evenly divided by gender and ranged in age from 8 to 46 years with the majority less than 18 years old (Table 1). The majority had SMA Type III and almost two-thirds were non-ambulatory. The 64 parents of individuals with SMA were predominantly female and their children with SMA ranged in age from 10 months to 20 years. Most had children with SMA Type II. Children of four of the parents were deceased due to complications of SMA. In two cases both parents of a child or a grandparent and a parent of a child participated together in a focus group. The 11 clinicians were predominantly male and had, on average, almost 21 years in practice. Most were pediatric neurologists and ten had practices that covered the SMA spectrum.

**Table 1:** Characteristics of the 96 study participants

*Characteristics*	*Number (%)*
*Individuals with SMA*	21
* Gender*	
Male	10 (48)
Female	11 (52)
* Age range (yrs.)*	
8–11	4 (19)
12–14	4 (19)
15–17	6 (29)
18–26+	7 (33)
* SMA type*	
Type I	1 ( 5)
Type II	8 (38)
Type III	12 (57)
* Currently ambulatory*	
Yes	8 (38)
No	13 (62)
* Form of participation*	
Interview	10 (48)
Focus Group	11 (52)
*Parents of individuals with SMA*	64
* Gender*	
Male	15 (23)
Female	49 (77)
* Age range of child with SMA (yrs)*	
<1 year	5 (8)
1–2 years	6(9)
3–11 year	30 (47)
12–17	19 (30)
18–25	5 (8)
* Mean age of diagnosis (yrs.)*	2.05
* SMA type*	
Type I	12 (19)
Type II	29 (45)
Type III	22 (34)
* Form of participation*	
Interview	21 (33)
Focus group	43 (67)
* Clinicians*	11
* Gender*	
Male	7 (64)
Female	4 (36)
* Care for multiple SMA types*	
Yes	10
No	1
* Years in practice (average, range)*	20.7, 12 -28
* Practice area/specialty*	
Physical therapy (neurological conditions specialty)	1 (9)
Pediatric physical therapy	2 (18)
Pediatric neurology	4 (36)
Pediatric orthopedic surgeon	1 (9)
Pulmonology	1 (9)
Neurosurgery	1 (9)
*Form of participation*	
Interview	7 (63)
Focus group	4 (36)

### Arriving at a diagnosis of SMA

The amount of time between the recognition of symptoms consistent with SMA and arriving at a diagnosis varied, but finding a diagnosis was a long process for many, requiring numerous visits to their pediatrician and specialists. Only those who lived close to medical centers with clinicians knowledgeable about SMA received a diagnosis relatively quickly, while others, particularly those who lived a long distance from large medical centers, endured weeks of testing and waiting for results. Below we outline three themes that characterized factors contributing to diagnostic delay.

#### Lack of awareness and knowledge about SMA

Although SMA is rare disease, it is common enough that larger pediatric practices could encounter a child with this disorder. One clinician in this study observed:

*…the incidence of the disease is such that every pediatrician is on average going to have one chance to blow the diagnosis in their career and unfortunately many of them take advantage of that opportunity* [3]

While this comment may overstate the prevalence of SMA, it emphasizes the point that most pediatricians are poorly prepared to identify SMA as a potential diagnosis among children who evidence symptoms.

The lack of knowledge among primary care clinicians, and even some neurologists, made it difficult for families to find a diagnosis. A parent of a child with Type III SMA described this challenge:

*…we spent 3–4 years trying to find a diagnosis and we didn’t have any luck so we were jumping from doctor to doctor… the local doctors, they didn’t have a clue of what could be wrong so we basically were struggling to find somebody to help us understand the problem* [11]

Parents often had to find information on their own or had to advocate for additional testing and assessment. A number reported that they succeeded in getting their children referred to a specialist only after they produced information from internet searches that might help to explain the symptoms of their children or pushed hard enough for additional assessments after being encouraged by friends or family to seek answers.

Not surprisingly, many parents were unaware of SMA prior to their child’s diagnosis, unless they had another child with this disorder. As a result, parents sometimes failed to recognize even significant deficits in motor function, a particular problem for first time parents with limited experience with infants. A clinician described being surprised when he found that the parents of a young infant were not aware of the profound abnormality of their son’s inability to move his limbs.

*… So I had a child not long ago whose parents at 3 weeks of age thought that child…had a problem with one hand because he couldn’t bring it up to the mouth and when I unwrapped the baby…he essentially, he couldn’t move anything except barely get one hand to the mouth and the other side was below that threshold. I was amazed that they didn’t imagine anything would be wrong because this is the first time they had ever had a baby and that they thought that’s the way babies were. So I stunned them with the information that there actually was something terribly, terribly wrong.* [3]

#### Distinguishing normal from abnormal development

One theme that emerged was the challenge of distinguishing normal from abnormal development in infants and young children, a distinction necessary to initiate the path to diagnosis. One clinician explained:

*The usual situation is just that there’s some fall off usually compared to either family members or peers in terms of motor function, and depending on whether its Type I, Type II, Type III, or whatever. It’s going to occur at different ages and as a consequence be demonstrated by age specific expectations. So the SMA1 kids maybe start seeming like they are gaining head control, but then they don’t fully gain it. Or they don’t develop the ability to roll over or whatever.* [4]

However, parents reported that they and the pediatrician often could not tell if the baby simply had a developmental delay because there is a large range of what is considered to be “normal” development. Commonly, parents of SMA Type I or II babies reported that they wondered if there was something wrong early on and brought up their concerns at regularly scheduled well-baby appointments, but the pediatrician was reluctant to refer them to a specialist. A parent of a child with Type II SMA recalled a common refrain: “’this is just developmental, come back and see me in two months’.” A parent of a Type I child described her experience with this problem:

*….So after one month I brought it up to a pediatrician and my son was 9 pounds and 2 oz when he was born, so our doctor said ‘well you know he’s a bigger baby, it’s sometimes harder for bigger babies to lift their heads simply because they’re heavier. So let’s give it one more month because of that’s within the typical range development…*[10]

Parents tended to defer to the judgment of the physician, and set aside their own fears, if only temporarily. One parent of a Type II child described how the pediatrician’s lack of information combined with their own inexperience resulted in a long period between the appearance of symptoms and arriving at a diagnosis.

*… So we didn’t have the best feeling but we were first time parents, and that’s what we’ve been told. You know, you’re first time parents, listen to what everyone else has to say and it will all be in time ok, but it wasn’t.* [37]

Parents of children with SMA Type III reported that their children appeared to develop normally until they reached an older age. Like their SMA Types I and II counterparts, they tended to ascribe early appearances of motor function deficits to natural differences between children. One parent recalled that when her son was fourteen he told her his hands were shaking and he had difficulty getting up off the floor. She reported,

“*He developed normally until about age ten or eleven, we noticed some differences but we were not concerned. We thought it was his unique body type and coordination.*” *[47]*

#### The challenges of differential diagnosis

Several parents of Type III children reported that their child showed signs of muscle weakness but the clinicians erroneously attributed these symptoms to other conditions, such as muscular dystrophy. One mother said:

*…the [local] neurologist…he didn’t actually understand exactly what was going on. His first impression or his only fear was that it could be muscular dystrophy.* [11]

Some children in this study had other chronic illnesses that masked the symptoms of SMA or they had been treated for other illnesses that led the parents or clinicians to attribute the symptoms of SMA to side effects of those medications, delaying a correct diagnosis. For example, one parent attributed her daughter’s weakness to the multiple medications required to treat kidney disease. The diagnosis of SMA was made when her daughter’s strength did not improve after stopping these medications.

#### Delivering the diagnosis

Parents reported a variety of ways that providers delivered the news about their child’s SMA diagnosis. Some complained that the clinician informed them of the diagnosis in a perfunctory phone call or short office meeting. Many of them felt that their doctors communicated the diagnosis in an insensitive and unhelpful manner. This style of communication was particularly hard for families when they were given news that was often unexpected and had weighty implications for their lives. A parent of a child diagnosed with Type II SMA described this experience:

*“She (the neurologist) looked at our son and evaluated him for probably no more than 3 minutes or less, and then said ‘please sit down. I’m 99 % sure your child has this condition and it’s terminal.’ So probably being in the room all of five minutes, they told us that our happy smiling gurgling child was going to die, and then she said ‘there’s nothing we can do.’ So we sat there in shock.”* [2]

Physicians, in comparison, spoke of the difficulty of communicating such “devastating news for the family,” especially because most parents have never heard of this condition.

*“If you get the diagnosis of spinal muscular atrophy, it’s definitely a devastating kind of news for the family. Particularly either Type 1 or 2 that you have to really tell the family the nature of the disease, the outcome, and many times it’s definitely impacting suddenly their whole lives… initially they cannot imagine what they will face in terms of care for a child or the impact of the disease. Not just for the child, but for the family, too. I think it’s a difficult diagnosis.”* [8]

### Views on Newborn Screening (NBS)

As shown in Table 2, five themes reflected positive views about NBS and three characterized worries. The most common, raised by parents across the three SMA types, was that NBS accelerated access to care, enabling them to connect with specialists and begin treatment as early as possible-earlier than they would have without the results of screening. In their view, starting physical therapy or nutritional and respiratory care as soon as possible was necessary to enhance a child’s abilities, minimize poor outcomes, and potentially prolong life.

**Table 2:** Parents’ views about newborn screening

Themes		Illustrative quotations
Positive	Early access to care	*… it would be helpful because if you found right away if SMA was present, then I think that you can be more proactive and start with all the therapies and whatever kind of nutrition would be needed for whatever type that you have* [9]
	Shorten time to diagnosis	*But some families they go to the doctor… their son, he’s not lifting his head ok we’ll give it another month, I think it’ll be okay give it another month, I guess I will do some testing, and sometimes their child has SMA type 1 and they don’t find out until they’re a year old. So I think that in those cases, in places that are not, that don’t have doctors that know about SMA, I think that it would be for raising awareness of SMA the screening, the newborn screening would be very beneficial.* [10]
	Prepare to care for a disabled child	*…. And also as a parent you’re preparing yourself both socially and psychologically to be ready for what’s to come ahead.* [9]
	Help first-time parents who may not know developmental milestones	*I think that’s a really wonderful idea because like I said the strongest thing for us was we were first time parents, we weren’t sure what was going on. …SMA is something we weren’t expecting and especially when the disease is so devastating, you know? It makes such a huge impact in your life, and the kid, not just a normal life but the mental health, physical, emotional, everything.* [26]
	Want information if it is available	*Well I’m always for access to more information in general; I think it should be an option, absolutely.* [44]
Negative	Prematurely affect my relationship with my child	*…I don’t know that I would have wanted to know and look at everything like ‘Oh no is that a sign of SMA?’* [47]
	Better not know in cases with very mild symptoms.	*On the other hand I suspect there are people that have SMA that…they almost never find out or they’re so much older when they find out. They were better off not knowing…I’m not sure* [7]
	Cost-benefit of population wide screening for a rare disease	*I think that I understand the reasoning why there is not newborn screening for SMA simply because it only effects 1 in 6,000 so I can understand that it’s very expensive to do for 1 in 6,000 babies then 5, 999 would not have it but I think that if we had all the resources in the world then absolutely.* [10]

Some parents explained that information from NBS would shorten the time it takes for a family to find a diagnosis for their child. This was particularly valuable given the challenges of diagnosing SMA. Parents of children with Type II SMA commented that receiving the results of NBS would be particularly helpful to first-time parents who may not know enough about expected developmental milestones to be able to recognize the symptoms of SMA early on.

Parents across the three SMA types believed screening results would help them to prepare to care for a child with physical disabilities. These included psychological preparations, finding support groups, as well as making preparations such as purchasing the necessary equipment, finding a home that is more handicapped-accessible, and making arrangements with insurance providers to insure coverage of extra expenses.

Fewer parents noted negative implications of NBS and those who did were primarily concerned that the results would negatively impact their relationship with their child. One parent believed that she would have been constantly vigilant, waiting for symptoms to appear if she learned of this diagnosis at her child’s birth. She likened this to waiting for a sign to appear as “*living on the precipice of something bad all the time”* and speculated… *“I don’t know that I would want that*.” Another worried that diagnosis prior to the onset of symptoms would take away her opportunity to have *“a normal time together where I just got to enjoy my child.*” [5] In a similar vein, a parent of a child with Type III wondered if there might be some individuals who have a mild form of SMA that would never be diagnosed and the results of NB screening would unnecessarily burden them with the knowledge that they have a very mild form of this disorder. One questioned the cost-benefit of screening for a rare disease.

### The psychosocial impact of SMA

The parents in this study described how the all-consuming nature of the demands of caring for a child with substantial physical needs affected the emotional and social lives of their entire family. As one parent said, *“It changes your whole life.”* [9] The never-ending nature of these demands took an emotional and a social toll on families including the siblings, as one mother explained:

*“For me it’s been real, real depressing. It’s taken a whole toll on my family. It is—I have to be able to accommodate to his needs without making my other two kids feel left out.”* [12]

Ten thematic areas characterized the psychosocial effects of living with SMA described by participants in this study. Illustrative quotations for each theme are in Table 3. Most of the areas affected both individuals with SMA and their parents although some were more relevant for parents, such as coming to terms with lost expectations for their children. All of the themes were pertinent across the three SMA types but like the SMA phenotype, the effects associated with each area lay on a continuum of severity, with the most severe typically described by parents of children with Type I SMA and the least severe by families and children with Type IIII SMA. One clinician described this:

**Table 3:** Thematic areas: psychosocial effects of life with SMA

Thematic areas	Illustrative quotations
Confronting premature death	*…we have a circle letter with 24 people and we know them all. So there’s constantly Type I’s dying in our circle and [my son] knows he has SMA so every now and then this will come up ….Jesus isn’t coming for me yet and I say no…* [51]
	*My fears are that my son’s going to die early.* [47]
	*…who’s just horribly depressed …is on anti-depressant medicines because he and he thinks about dying and all of that*. [5]
Difficult choices	*My husband and I chose not to do any invasive treatment with our daughter, that’s why she died so soon. She did have a g tube, a feeding tube. We did use a suction machine, we rarely used an oxygen tank. Other than that, nothing more, so she had a lot of trouble breathing and ultimately, that’s what took her life. But for us, it was quality over quantity. And I had heard from many other families who had Type 1 s, Type 2 s that have used trachs and things like that, and it just wasn’t something that we wanted for our child. So, we made those decisions because of that.* [49]
	*…they said, ‘Our options are palliative-type hospice care,’ which basically means turn off the machines, that’s the end, ‘or we can give him a trach.’ Our family decided that’s the route we were going to take. We gave him a trach. We gave him a gastric feeding tube. We really didn’t know his future. We knew it would be a journey. We knew that our lives would change forever. …Our house is like a miniature NICU.* [43]
Heartbreak and fear with loss of functional abilities	*It’s frightening, and it’s so cruel because it declines and then you’ll plateau for a little while and every time you plateau, you start to feel like ok, maybe we can hang out here for just a while but the scary thing is you never know when the next decline is coming. So every day is just a fear of will today be the day and plus, every time he loses some ability or every time his respiratory health declines, we always wonder what else he has to lose.* [30]
	*…and sometimes it’s like oh screw it, I can’t do this anymore. You know, like the feeding myself. For a while, I’d bring blocks that I’d put under the table at a restaurant to raise the table up, things like that. And then it got to the point where I was struggling so much to eat, I’d choke more often and then having people feed me, I started to choke more often. You know, like oh crap, I got to give this up* [31]
Coming to terms with lost expectations	*…you’re basically dying to all those expectations that you had had… my son was the thing that brought me such joy, you know, the person in my life that makes me feel happy, but then also looking at him reminded me of the disease is the thing in our life he says that makes us unhappy. So it was hard to balance, you know, still being a great mom and still encouraging and loving at the same time of grieving. So you know I gave myself time every day to be sad to try and do it away from my son.* [10]
Loss of sleep and stress	*He’s very dependent on me, and it’s driving me crazy…He won’t let his father or anybody else take him to the bathroom or change him or feed him or anything. That drives me crazy personally because I never get a break.* [12]
	*…You have to roll her over, we have to place her arms where they’re going to be when she falls asleep,” explains the mother of a Type 2 patient. “Her head, her legs exactly how she wants them, and then she sleeps for a while, she wakes up, calls, and you go in and roll her over and place everything again….(we have to move her) every hour to an hour-and-a-half.* [14]
Social discomfiture & stigma	*…he needs to read “The Great Gatsby” over the summer so we were in this little bookstore. …He walks in and the man’s going ‘no, I don’t have it, oh, but get up, step up on this crate and get that book up there that might have something.’ He (son) can’t do that. But the person didn’t realize it, so I got up and got it, but we didn’t go into an explanation why I had to do it. And so that stuff, you know, you’re not going to grieve, but there’s a loss there. Can’t step up on stool to get something, and you feel a little awkward when you’re as able-bodied as you look, but yet something as simple as that you can’t do.* [9]
	*I mean it’s hard to go out with a disability; it’s hard to make friends with a disability. At least now that I’m not in a wheelchair I can kind of hide it, and it’s not as obvious… People treat you differently when you’re in a wheelchair.* [19]
Limitations on social activities	*That was a lot, it was extremely overwhelming. Especially because I wanted to take her outside, I wanted to go on a walk; she was inside so much that it just wasn’t fair. It was difficult to move her because she would stop breathing, just to change her diaper she would stop breathing, so it was really hard. I think once or twice I had tried to take her out on my own; we lived in an apartment complex at that time that had a beautiful park. I wanted to take her walking and I tried and she had a blue spell, and I had to somehow carry (her) inside. Now she’s deadweight at this point and she was actually very, very long for her age so it was a lot to carry her and I would always carry a morphine syringe filled with morphine anywhere I went because she would just stop breathing. And just trying to do all that and manage the keys, just doing all that stuff all by myself.* [49]
	*I actually went to a festival over the weekend and I needed to use a handicapped bathroom, and the only handicapped accessible bathrooms in the whole entire place were for VIP. It’s like, ok, but I can’t use the stairs. They couldn’t get it through their heads that they need a handicap accessible bathroom for everyone…Even my friends will be like do you want to come to this party and it will be late at night, and I’m like, I already went out today, I’m too tired.* [19]
Struggle to achieve independence	*… he’s 17 in two months, he goes off to college in two years, that’s my hope. I fear he’s going to have to have a parent at college just because there’s many things that he can’t do by himself.* [47]
	*…I’m already worried about finding a job right now without a wheel chair…*[19]
Uncertainty & Helplessness	*The burden, I guess as parents, is just feeling helpless. Knowing that your child has a disease that there’s really not much you can do about, and the options you do have are very, very scary and very invasive. And no matter what, the disease will progress. I guess that was the initial burden, just knowing that this was going to happen and it was basically a train wreck that you had no control over.* [49]
	*…when you start this journey you really don’t think your child is going to live, and planning a funeral now for 13 years as well as you know saving for a college fund. So it’s a very bizarre mind trip that you’re going through. But when majority of cases with SMA are Type 1 and unfortunately so many children do die, but when you’re going along on this journey as a parent you don’t know what to plan for. We were in a tri-level home with a boy in a wheelchair and no bedroom on the downstairs level, no bathroom, we didn’t know if he was going to live though. If he wasn’t going to live do we move or do we not, you know. It wasn’t until he was 6 we moved to a single story home. You don’t know how to plan.* [2]
Pressure on Family Finances	*…At the beginning I took maternity leave, but I got some sort of compensation for that, but at the end, no. I was very fortunate I have an employer that was extremely flexible and understanding. Not everybody’s like that, and I can imagine that some people would lose their job, or their position or something would happen. I can’t imagine, especially with a sick child.* [49]
	*SMA children are not cognitively delayed, they’re just physically fragile. So that really leaves a gap in care, because most funding sources require children to be both cognitively and physically disabled. That’s not the case with a neuromuscular disease. Even though they need a lot of care, they’re not cognitively delayed. So that means that this population is under, they’re lacking a safety net. … [state] does not support SMA families at all. And that’s a real problem. Because without their support, the families are left to struggle, to leave their job to take care of their child.* [33]

*Again it depends on the level of severity of the disease, so in the more mildly affected the impact can be fairly minimal. The oldest patient I have was 84 when she walked into my office. The impact on that family was almost non-existent. For SMA1 families the impact is really quite profound.* [4]

#### Confronting premature death

Facing premature death is one of the most unimaginably challenging outcomes of this condition for families and individuals with SMA. This was not unique to Type I SMA; for example a mother of a child with Type III SMA expressed her fears about early death. A clinician also described treating an adolescent with SMA Type III who was depressed and thinking about death.

#### Difficult treatment choices

Families and clinicians described the difficult treatment choices that some families faced, such as whether to pursue an invasive treatment regimen for a child whose respiratory function is failing. Based on their family’s values, a family might choose palliative care with no invasive treatments, some invasive treatments, or they might pursue all available treatment options. One family in this study did not elect to use respiratory support for the baby with Type I SMA because they feared that their child’s quality of life would suffer unacceptably. Others chose to utilize all measures available, even though they realized that they could not be certain about what their child’s future might hold, and that caring for their Type I child would be a major undertaking for the entire family.

#### Heartbreak and fear with loss of functional abilities

Parents and individuals with SMA described living with the constant fear of diminished physical function as the condition progressed and the sadness associated with these losses. Parents said that watching their children decline was terribly difficult emotionally. For example, one described the heartbreak of watching her son lose his ability to smile, laugh and communicate joy. An adult patient talked about the difficulty of learning to accept her losses and giving up activities such as eating out at a restaurant with friends.

#### Coming to terms with lost expectations

Parents talked about coming to terms with lost expectations for their children and experiencing *“a lot of grief a lot of letting go of things that I had hoped for my child”* [7] after learning about the SMA diagnosis and realizing their child would never be like other children who do not live with a deteriorating condition. For one parent this meant learning to deal with conflicting feelings of deep love for her child and the sadness brought to her life because of his condition.

#### Loss of sleep and stress

Loss of sleep and the never-ending burdens of caring for a child with substantial physical disability were a concern. Many children required high levels of physical care and constant supervision, day and night. Some described how these physical demands became increasingly difficult to manage as the child grew in size. Many parents had to awaken multiple times during the night to help their child rollover to prevent bedsores, or even to simply adjust the covers to prevent the child from getting too hot or cold.

#### Social discomfiture and stigma

In addition to facing the daily challenges of their children having a physical disability, individuals with SMA experienced social discomfort and stigma such as their embarrassment when they could not perform physical activities. Some patients described their frustration with lack of handicapped accessible services, and frustration with being stereotyped and treated differently than others without a physical disability.

#### Limitations on social activities

Both parents and patients described how they were limited in their ability to socialize and engage in activities outside of their homes for several reasons. Some children required a lot of equipment to support their breathing or movement making preparation for activities outside of the home time consuming and overwhelming. A family of a child with milder disabilities described how they loved to hike but had to stop when their daughter required a wheelchair. Others described how the need to protect their child from respiratory infections required them to limit outdoor activities or even keep their young children out of school. Finally, patients were frustrated by lack of handicapped access or limited ability to socialize because of weakness and fatigue.

#### Struggle to achieve Independence

Achieving independence was a worry for parents and individuals with SMA. For example, parents who wanted their children to experience an independent life as adults had to weigh this heavily against the reality of how much assistance their child would need away from home and what that assistance would cost. The mother of a 17-year-old with Type II SMA was proud of her daughter, who excelled in school and was applying to college, but she worried about getting her the supportive care she would need to aid her to dress, toilet, and shower while away at school.

#### Uncertainty and helplessness

Parents said that in living with SMA they felt very helpless and out of control, especially regarding the future. They noted how the uncertain trajectory of the decline in a child’s functional status or even life expectancy made it difficult for them to plan ahead. One parent explained the painful paradox of planning for a child to die while planning for his or her future.

#### Pressure on family finances

Several participants described the financial impact of having a child with SMA. A clinician estimated that the cost of raising a child with a degenerative neuromuscular disease was “in the millions, per child.” A mother described the financial burden of lost income as a result of taking time off from work or needing to re-arrange a work schedule to accommodate the extra needs of their child. Some parents experienced problems with obtaining adequate support for their Type III children who attended public schools. For example, several noted that some states did not offer services for children who have physical disabilities without cognitive impairment. In some instances this forced families to make other arrangements for schooling, which sometimes required leaving their jobs.

## Discussion

The results of this study augment the existing knowledge about the journey to SMA diagnosis, views on NBS, and the impact of SMA on those living with this condition. The findings underscore the broad range of this impact and the high level of burden families experience, and the challenges individuals face in coping with this disorder.

Our findings highlighted two important challenges for families seeking a diagnosis. First, the process can be long and difficult and delays hamper prompt access to treatment or participation in clinical trials [2]. Improving the speed of diagnosis can mean earlier initiation of treatment and help individuals with SMA Types II and III better maintain motor function. Similarly, earlier diagnosis of infants with SMA Type I means that interventions can be initiated before a child becomes nutritionally compromised and develop neurologic and respiratory damage [11, 32]. In addition, earlier diagnosis can help parents find the resources they need more quickly and better support their financial, social and psychological preparations to care for a child with significant physical disability.

A second challenge of the diagnostic process for families is that the emotional effects of a prolonged diagnostic journey are exacerbated when clinicians deliver the diagnosis in an insensitive manner. These findings support those reported by Lawton and her colleagues [20] about an Australian study and, to the best of our knowledge, the only other study to examine families’ experiences in arriving at a diagnosis of SMA.

Difficulties with the diagnosis stem, in part, from lack of awareness and knowledge about SMA and educating parents, pediatricians and other clinicians about SMA may help to alleviate delays. Studies by Rothwell [32] and Meldrum [15] point out the need for better education of parents about SMA both prior to genetic testing and at the time of an SMA diagnosis. In addition, pediatricians would benefit from increased awareness about this disorder in general as well as training on how to deliver news of a diagnosis of a life altering condition such as SMA in a less negative, and more sensitive and knowledgeable manner. Communicating about symptom progression is difficult for any clinician talking with patients about a serious illness, but it is even more challenging when discussing a condition that is virtually unknown by the general public and where the symptom trajectory is unpredictable, as it is with SMA.

The finding that most parents supported the addition of SMA screening to universal NBS panels is consistent with other studies. In a survey of parents of children with neuromuscular diseases and unaffected expectant parents, over 90 % of the participants supported NBS for these conditions, including SMA [33]. In a qualitative study on SMA NB screening, participants viewed screening to be of low risk and with potential benefit but they felt that parents should have the opportunity to opt-out of the screening [32].

In the United States, specific guidelines and processes have been established by the Federal government for adding diseases to the Recommended Uniform Screening Panel ( See http://www.hrsa.gov/advisorycommittees/mchbadvisory/heritabledisorders/index.html for details). It is important to note, however, that expanding these panels raise ethically complex questions. Recommendations to include SMA in this panel should be considered in light of three caveats. First, NBS information cannot definitively predict the severity of the phenotype, the age of onset of symptoms, or the trajectory of their child’s condition. However, in the specific case of SMA, SMN2 copy number forecasts this, albeit not perfectly [34] Second, approved pharmacological treatments are not available at this time. When such treatments are available screening will become critical in order to treat patients as early as possible, which has been shown to be beneficial in mouse models of SMA [35] Currently, the principal benefit of adding of SMA screening is to shorten the time to diagnosis and to begin proactive clinical care, which itself has been shown to alter the natural history of type I SMA [36, 37]. Third, the decision to add SMA screening to NBS panels must also be weighed against potential detriment to parents’ experiences with their newborn prior to onset of symptoms, a concern raised by a few parents in this study. However, as discussed previously, previous survey results about parental attitudes about NBS for SMA have been very positive [15, 32].

The importance of understanding the emotional and social effects of caring for a child with a disabling physical condition like SMA cannot be overemphasized. Families and individuals with SMA face multi-faceted challenges including feelings of loss and helplessness, limitations on activities outside of the home, and financial concerns. Experienced alone, each of these effects can have a significant impact on the quality of life of an individual or family. Combined, they can seem insurmountable and sometimes uncontrollable. The psychological impact for individuals with SMA and their families is complicated by the fact that the condition is progressive. Parents and individuals with SMA described the painful juxtaposition of waiting for functioning to decline while struggling and hoping to maintain function. Uncertainty about the trajectory of the condition made coming to terms with this condition more challenging than it might be otherwise.

Our findings are consistent with the reports of lower quality of life reported by 35 families and children surveyed in the Czech Republic [21], reports of higher rates of stress and strain and lower rates of social supports reported by parents of children with SMA in Germany [38] and lower scores on the Pediatric Quality of Life Inventory among 176 children with SMA compared to healthy children [39]. A study of 37 adults with SMA II who lived independently in Denmark found that these individuals successfully managed their lives although they lived with anxiety about the future and feared progressive decline in their motor function [40]. This suggests that individuals with SMA can successful lives given adequate supports such as those offered to families in Denmark.

It is important to keep in mind that just as the clinical features of this condition are highly variable, so are the psychosocial effects of living with this rare disease [16]. For example, without effective treatment, parents of Type I children can face choosing between invasive treatments to keep their child alive past the first year or palliative care. On the other end of the continuum, parents of Type III children experience the stress of letting go of their need to protect their child and instead prepare them to live as independently as possible.

While our study sample was relatively small (21 patients, 64 parents, 11 clinicians), semi-structured interviews allowed us to obtain in-depth information about the experiences of individuals living with SMA as expressed in their own words. This is especially important given the psychosocial nature of many of our research questions. Our study offers a clearer understanding of the patient journey to diagnosis across SMA types and identifies at least two opportunities to shorten the diagnostic delay, i.e., educating clinicians to promote earlier diagnosis and offering SMA newborn screening for those parents who want it. In addition, this study enhanced our understanding of complexity of this condition as well as its psychosocial impact on individuals with SMA and their parents. Several topics emerged which deserve broader investigation using quantitative methods. For example, a study involving a larger sample of families and children affected by SMA would provide more specific information about the factors that affect the diagnostic journey, access to supports, and family coping.

The experiences and views described by the participants in this study reflect a self-selected sample recruited through the SMA advocacy groups and may not reflect the views of those not connected to these organizations. Finally, because the majority of participants were parents of individuals with SMA, our findings emphasize their perspective more than the perspective of individuals with SMA and clinicians who care for them.

## Conclusions

SMA presents complex challenges for individuals with this condition and their families. Researchers and clinicians have invested substantial effort into understanding the genetic basis of this inherited disorder; its clinical features and their management. Less attention has been paid to understanding the effect on the lives of families and individuals with SMA across the disease spectrum. The results of this study reveals the considerable psychosocial impact of this condition beginning with the difficulties of the diagnostic process and views on how newborn screening might ameliorate the process and suggests important unmet needs for early diagnosis and effective treatment.

## Appendix

### Questions Covered in SMA Focus Groups and Interviews

1. Let’s start by talking a little about what was happening from the time when [YOU/YOUR PATIENT/THE PERSON YOU CARE FOR] first had symptoms to when they finally got diagnosed. How long did that take and what was that like?
2. What has been the impact of SMA?
3. Do you think newborn screening of SMA would be helpful?
4. What does it feel like for someone who might have this condition?
  - [PROBE] What might someone who has this condition experience?
5. Let me go up to the board, and let’s list some of the ways that SMA [YOU/YOUR PATIENT/THE PERSON YOU CARE FOR].
6. [FOR EACH LISTED] How big of an impact has this had on [YOUR LIFE/YOUR PATIENT’S LIFE/THE LIFE OF THE PERSON YOU CARE FOR]?
7. [IF NOT MENTIONED] Do [YOU/YOUR PATIENT/THE PERSON YOU CARE FOR] do things/take steps to avoid being hospitalized? Tell me about that?
8. Do [YOU/YOUR PATIENT/THE PERSON YOU CARE FOR] do things/take steps to avoid [RES-PIRATORY EVENTS/THINGS THAT MIGHT IMPACT BREATHING]? Tell me about that?
9. In the past 12 months, what kind of decline in motor function did [YOU/YOUR PATIENT/THE PERSON YOU CARE FOR] experience?
10. Would a treatment that stops decline in motor function be meaningful to [YOU/YOUR PATIENT/THE PERSON YOU CARE FOR]?
11. Would a treatment that improves your motor function be meaningful to [YOU/YOUR PATIENT/THE PERSON YOU CARE FOR]?
12. What kind of improvement do [YOU/YOUR PATIENT/THE PERSON YOU CARE FOR] want to see from treatment?
13. Take a look at [CORRESPONDING ITEM FROM HAMMERSMITH SCALE]. Would a partial im-provement (i.e. 0 to 1 or 1 to 2) be meaningful?
14. What other changes from treatment be meaningful to [YOU/YOUR PATIENT/THE PERSON YOU CARE FOR]?

## Abbreviations

SMA: Spinal muscular atrophy
SMN1: Survival motor neuron 1 gene
NBS: Newborn screening
HFMSE: Hammersmith functional motor scale expanded
